# Interaction of Hydroxychloroquine with Pharmacokinetically Important Drug Transporters

**DOI:** 10.3390/pharmaceutics12100919

**Published:** 2020-09-25

**Authors:** Johanna Weiss, Gzona Bajraktari-Sylejmani, Walter E. Haefeli

**Affiliations:** Department of Clinical Pharmacology and Pharmacoepidemiology, Heidelberg University Hospital, Im Neuenheimer Feld 410, 69120 Heidelberg, Germany; gzona.bajraktari-sylejmani@med.uni-heidelberg.de (G.B.-S.); walter.emil.haefeli@med.uni-heidelberg.de (W.E.H.)

**Keywords:** hydroxychloroquine, drug-drug interaction, drug transporters, inhibition, induction, P-glycoprotein, BCRP, OATP

## Abstract

(1) Background: Hydroxychloroquine is used to treat malaria and autoimmune diseases, and its potential use against COVID-19 is currently under investigation. Thus far, information on interactions of hydroxychloroquine with drug transporters mediating drug-drug interactions is limited. We assessed the inhibition of important efflux (P-glycoprotein (P-gp), breast cancer resistance protein (BCRP)) and uptake transporters (organic anion transporting polypeptide (OATP)-1B1, OATP1B3, OATP2B1) by hydroxychloroquine, tested its P-gp and BCRP substrate characteristics, and evaluated the induction of pharmacokinetically relevant genes regulated by the nuclear pregnane X (PXR) (*CYP3A4, ABCB1*) and aryl hydrocarbon receptor (AhR) (*CYP1A1, CYP1A2*). (2) Methods: Transporter inhibition was evaluated in transporter over-expressing cell lines using fluorescent probe substrates. P-gp and BCRP substrate characteristics were assessed by comparing growth inhibition of over-expressing and parental cell lines. Possible mRNA induction was analysed in LS180 cells by quantitative real-time PCR. (3) Results: Hydroxychloroquine did not inhibit BCRP or the OATPs tested but inhibited P-gp at concentrations exceeding 10 µM. P-gp overexpressing cells were 5.2-fold more resistant to hydroxychloroquine than control cells stressing its substrate characteristics. Hydroxychloroquine did not induce genes regulated by PXR or AhR. (4) Conclusions: This is the first evidence that hydroxychloroquine’s interaction potential with drug transporters is low, albeit bioavailability of simultaneously orally administered P-gp substrates might be increased by hydroxychloroquine.

## 1. Introduction

Hydroxychloroquine is preferred over its analogue chloroquine due to its lower risk for ocular toxicity [1,2]. Initially developed against malaria, it is also widely used against several autoimmune diseases such as rheumatoid arthritis or systemic lupus erythematosus (SLE) [3,4,5]. Moreover, it also exerts anti-bacterial, anti-fungal and anti-viral effects, which came to the fore in 2020, when treatment options against SARS-Cov-2 were evaluated [3,5,6,7]. Similar to chloroquine, hydroxychloroquine is thought to be metabolised via the cytochrome P450 (CYP) system, presumably via CYP2C8, CYP3A4, and CYP2D6 [6,8], but there is no definite data available proving this assumption. Only for CYP2D6 there is data indicating that this enzyme is involved in hydroxychloroquine metabolism and that it is inhibited by hydroxychloroquine [9,10].

Beyond drug-metabolising enzymes, drug efflux or uptake transporters can also critically influence the pharmacokinetics of drugs. Iatrogenic enhancement or suppression of drug transporter activity can lead to decreased or increased exposure of concurrently administered drugs reducing their efficacy or increasing the risk for adverse effects [11,12,13,14]. Two of the most important drug efflux transporters mediating drug-drug interactions are P-glycoprotein (P-gp) and breast cancer resistance protein (BCRP) [11,13]. For instance, P-gp mediates several clinically relevant interactions through its enzymatic inhibition (e.g., quinidine or clarithromycin) or transcriptional induction (e.g., rifampicin) [11,13]. On the other side, members of the organic anion transporting polypeptide (OATP) family are important uptake transporters being implicated in many drug-drug interactions. For example, inhibition of hepatic OATP1B1/OATP1B3 transporters or intestinal OATP2B1 transporters mediate several food- or drug-drug interactions including the well-known interactions with statins [11,13,15].

So far, there is only sparse information on possible interactions of hydroxychloroquine with drug transporters. Limited available evidence suggests that hydroxychloroquine might be an inhibitor of P-gp because it can increase the serum concentrations of the P-gp substrate digoxin [16], but the underlying mechanism of this interaction has not been clarified yet.

We, therefore, aimed to assess hydroxychloroquine’s inhibitor potential of important efflux (P-gp and BCRP) and uptake transporters (OATP1B1, OATP1B3, and OATP2B1), which frequently mediate drug-drug interactions. Secondly, we evaluated possible P-gp and BCRP substrate characteristics, which could have an impact on the pharmacokinetics of hydroxychloroquine itself. Thirdly, we examined possible inducing properties of hydroxychloroquine on transporter genes. Because most inductions are mediated by the nuclear pregnane X receptor (PXR) and some by the aryl hydrocarbon receptor (AhR), we focused on transporter genes regulated by these transcription factors (*ABCB1* (encoding for P-gp) and *ABCG2* (encoding for the breast cancer resistance protein, BCRP)) and included some CYP genes in this analysis, which serve as prototypical indicators for PXR-regulated (*CYP3A4*) and AhR-regulated genes (*CYP1A1* and *CYP1A2*).

## 2. Materials and Methods

### 2.1. Materials

Cell culture media, supplements, Hank’s balanced salt solution (HBSS), HEPES, phosphate-buffered saline (PBS), foetal calf serum (FCS), tetracycline, omeprazole, naringin, rifampicin, 4′,5′-dibromofluorescein (DBF), fumitremorgin C (FTC), the Cytotoxicity Detection Kit (LDH), and the GenElute™ Mammalian Total RNA Miniprep Kit were purchased from Sigma-Aldrich (Munich, Germany). The RevertAid™ H Minus First Strand cDNA Synthesis Kit and the Absolute QPCR SYBR Green Mix were purchased from Thermo Fisher Scientific (Waltham, MA, USA) and dimethyl sulfoxide (DMSO), Triton X-100, and crystal violet were from AppliChem (Darmstadt, Germany). Calcein acetoxymethylester (calcein-AM) was purchased from Invitrogen (Karlsruhe, Germany), pheophorbide A from Frontier Scientific Europe (Carnforth, UK), and 8-fluorescein-cAMP (8-FcA) from BIOLOG Life Science Institute (Bremen, Germany). LY335979 (zosuquidar) was obtained from Toronto Research Chemicals (Toronto, ON, Canada) and hydroxychloroquine from Cayman Chemical (Ann Arbor, MI, USA). Primers were synthesised by Eurofins MWG Operon (Ebersberg, Germany).

### 2.2. Cytotoxicity Assays

The toxic effects of tested compounds can negatively influence transporter inhibition assays. We, therefore, tested hydroxychloroquine for its possible cell-toxic effects using the Cytotoxicity Detection Kit (LDH) that indicates the release of lactate dehydrogenase (LDH). Hydroxychloroquine had no toxic effects up to 100 µM in all cell lines used.

### 2.3. P-gp Inhibition Assay (Calcein-AM Uptake Assay)

P-gp inhibition was tested with a calcein assay as published previously [17,18] using two different cell systems: the L-MDR1 cell line that over-expresses human P-gp and the corresponding parental cell line LLC-PK1 [19] and the P-gp over-expressing murine monocytic cell line P388/dx [20] and its parental counterpart cell line P388 as a control. LLC-PK1 and L-MDR1 cells were cultured under standard cell culture conditions with medium M199 supplemented with 10% FCS, 2 mM glutamine, 100 U/mL penicillin, and 100 µg/mL streptomycin sulphate. To maintain P-gp expression, the culture medium for L-MDR1 was supplemented with 0.64 µM vincristine. One day before using the cells for the calcein assay, both cell lines were fed with vincristine-free culture medium. P388 cells were cultured under standard cell culture conditions with RPMI 1640 medium supplemented with 10% FCS, 2 mM glutamine, 500 mM β-mercaptoethanol, 100 U/mL penicillin, and 100 µg/mL streptomycin sulphate. For maintaining P-gp expression, the culture medium for P388/dx contained 0.43 µM doxorubicin. One day before the inhibition assay, both P388 cell lines were set to the doxorubicin-free culture medium.

In brief, the assay was performed in 96-well plates with a final calcein-AM concentration of 0.5 µM for the LLC cell system and 1 µM for the P388 cell system. Cells were washed with pre-warmed HBSS supplemented with 10 mM HEPES (HHBSS) and pre-incubated with this buffer for 30 min and subsequently with the test compound for 10 min (LLC cell system) or 15 min (P388 cell system). After pre-incubation, calcein-AM was added and the cells were incubated for 60 min (LLC system) or 30 min (P388 cell system) at 37 °C on a rotary shaker (Noctua, Vienna, Austria). The corresponding time points were chosen based on the highest possible measurement range for inhibition. The uptake was stopped by transferring the plates on ice and washing the cells twice with HHBSS pre-cooled to 4 °C. Subsequently, cells were lysed in 1% Triton X-100 for 15 min. The fluorescence of the calcein generated within the cells was analysed in a Fluoroskan Ascent fluorimeter (Labsystems, Frankfurt, Germany) with 485 nm excitation and 535 nm emission filters. Each concentration of hydroxychloroquine (0.01–100 µM) was tested in octuplet and the experiment was performed in quadruplicate. 200 µM verapamil was used as a positive control. Possible quenching effects of hydroxychloroquine were ruled out because no effect on calcein fluorescence was observed in the parental cell lines.

### 2.4. BCRP Inhibition Assay (Flow Cytometric Pheophorbide A Efflux Assay)

BCRP inhibition was tested by using pheophorbide A as a fluorescent substrate probe in BCRP over-expressing MDCKII-BCRP cells [21] and its parental counterpart cell line MDCKII. Both cell lines were cultured under standard cell culture conditions in DMEM containing 10% FCS, 2 mM glutamine, 100 U/mL penicillin, and 100 µg/mL streptomycin sulphate. The assay was conducted as described previously [22]. In brief, 10^6^ cells were suspended in 400 µL incubation medium (RPMI/2% FCS) containing 1 µM pheophorbide A and incubated at 37 °C on a rotary shaker (Noctua, Vienna, Austria) for 30 min. After washing the cells with an ice-cold incubation medium, cells were re-suspended in a medium containing the respective test or control compound and incubated at 37 °C on a rotary shaker for 60 min. This time point was chosen based on the highest possible measurement range for inhibition. After washing the cells with an ice-cold incubation medium, they were re-suspended in 500 µL ice-cold PBS/2% FCS and measured immediately. Intracellular fluorescence was quantified in a MACSQuant 10 Analyzer (Milteny, Bergisch-Gladbach, Germany) with the 488 nm laser and the 525 nm bandfilter for GFP (FITC channel) and the 638 nm laser and the 650 nm bandpass filter for pheophorbide A (APC channel). In each sample, 30,000 cells were counted. Excluding debris, living cells were gated in the forward/sideward scatter by using the unstained sample. BCRP-positive cells also express a green fluorescent protein (GFP) [21] and thus, BCRP-positive cells were additionally gated in the FITC channel. The inhibitory effect of hydroxychloroquine and the positive control compound (10 µM FTC) was quantified by calculating the ratio between the median fluorescence with and without the inhibitor and normalising the effects to those measured in the parental cell line. The experiment was performed in triplicate on different days. Possible quenching effects of hydroxychloroquine were ruled out because no hydroxychloroquine effect on pheophorbide A fluorescence was observed in the parental cell line MDCKII.

### 2.5. Flow Cytometric OATP1B1 and OATP1B3 Inhibition Assay

As cell models for human OATP1B1 and OATP1B3, HEK293, a human embryonic kidney cell line, stably transfected with OATP1B1 (HEK-OATP1B1), OATP1B3 (HEK-OATP1B3), or the empty control vector (HEK293-VC G418) were used [23,24]. Cells were cultured under standard cell culture conditions with DMEM supplemented with 10% FCS, 2 mM glutamine, 100 U/mL penicillin, 100 µg/mL streptomycin sulphate, and 800 µg/mL G418. Inhibition of the OATPs was quantified by flow cytometry assessing the uptake of 8-FcA into HEK293 cells over-expressing the respective transporter after 10 min incubation and normalised to the control cell line as described previously [25]. Intracellular fluorescence was quantified in a MACSQuant 10 Analyzer (Milteny, Bergisch-Gladbach, Germany) with the 488 nm laser for excitation and the 525 nm bandfilter for emission (FITC channel). Excluding debris, living cells were gated in the forward/sideward scatter by using the unstained sample. For each concentration (0.1–100 µM), 30,000 cells were measured and each experiment was performed in triplicate. Rifampicin (20 µM), a well-known inhibitor of OATP1B1 and OATP1B3, was used as a positive control. Possible quenching effects of hydroxychloroquine were ruled out because no effect on 8-FcA fluorescence was observed in the parental cell lines.

### 2.6. Flow Cytometric OATP2B1 Inhibition Assay

As a cell model for human OATP2B1, HEK293 cells over-expressing OATP2B1 in the presence of tetracycline were used [26]. Cells were cultured under standard cell culture conditions with DMEM/Ham’s F12 medium supplemented with 10% FCS, 4 mM glutamine, 100 U/mL penicillin, and 100 µg/mL streptomycin sulphate. To generate OATP2B1 over-expression, cells were treated 72 h before the assay with 1 µg/mL tetracycline.

The inhibition of OATP2B1 was analysed by using the fluorescent substrate DBF (1 µM). Intracellular fluorescence was quantified after 10 min of incubation in a MACSQuant 10 Analyzer (Milteny, Bergisch-Gladbach, Germany) with the 488 nm laser for excitation and the 525 nm bandfilter for emission (FITC channel). Excluding debris, living cells were gated in the forward/sideward scatter by using the unstained sample. For each concentration (0.1–100 µM), 30,000 cells were measured and each experiment was performed in triplicate. Uptake in cells with tetracycline induction was normalised to the uptake in cells without induction. Naringin (1 mM) was used as a positive control. Possible quenching effects of hydroxychloroquine were ruled out because no effect on DBF fluorescence was observed in the control cell line.

### 2.7. Growth Inhibition Assays with Resistant Cell Lines for Assessing P-gp and BCRP Substrate Characteristics

To evaluate whether hydroxychloroquine is a substrate of P-gp or BCRP, growth inhibition assays were conducted. Cell lines with overexpression of an efflux transporter are more resistant to the anti-proliferative effects of a compound if this compound is a substrate of the respective transporter and can be extruded from the cell. Thus, anti-proliferative effects of hydroxychloroquine were assessed in L-MDR1 cells (with over-expression of human P-gp) in comparison to the effects in the parental cell line LLC-PK1 and in MDCKII-BCRP cells (over-expressing human BCRP) compared to the parental cell line MDCKII. Reversal of drug resistance was tested by using the P-gp-specific inhibitor LY335979 (zosuquidar) or the BCRP-specific inhibitor FTC. The assay using crystal violet staining was conducted and analysed as described previously [27]. Each experiment was performed at least in quadruplicate with *n* = 8 wells for each concentration (0.1–500 µM).

### 2.8. Induction Assay

For induction experiments, the human colon adenocarcinoma cell line LS180 (available at ATCC, Manassas, VA, USA) was used. This cell line is an established model for investigating gene inductions mediated by PXR or AhR [25,28]. Cells were cultured under standard cell culture conditions in DMEM supplemented with 10% FCS, 100 U/mL penicillin, 100 µg/mL streptomycin sulphate, 0.1 mM non-essential amino acids, and 2 mM glutamine.

To exclude an anti-proliferative effect potentially flawing the results of the induction assay, growth inhibition by hydroxychloroquine was investigated in LS180 cells beforehand. Proliferation was quantified by crystal violet staining and the assays were conducted as described previously [27]. Each concentration was tested in octuplet and each experiment was performed in quadruplicate. The IC_20_ for hydroxychloroquine was 22.5 ± 7.3 µM. Thus, to ensure that > 80% of the cells survive, the maximum concentration tested was set to 20 µM.

For the induction assays, LS180 cells were treated for four days with hydroxychloroquine (1, 2, 10, and 20 µM), rifampicin (20 µM, positive control for PXR-driven genes), omeprazole (150 µM, positive control for AhR-driven genes), and compound-free medium as a negative control, respectively. All media were adjusted to the DMSO content of the solution with the highest DMSO content in the respective experiment (0.1%). The experiment was conducted in quintuplicate. RNA was extracted immediately after harvesting the cells and stored at −80 °C until processing.

RNA was isolated using the GenElute™ Mammalian Total RNA Miniprep Kit, and cDNA was synthesized with the RevertAid™ H Minus First Strand cDNA Synthesis Kit according to the manufacturer’s instructions. mRNA expression was quantified by real-time RT-PCR with the LightCycler^®^ 480 (Roche Applied Science, Mannheim, Germany) as described previously [29]. Primer sequences are listed in Table 1. PCR amplification was carried out in 20 µL reaction volume containing 5 µL 1:10 diluted cDNA, 1× Absolute QPCR SYBR Green Mix and 0.15 µM (*ABCB1, ABCG2, CYP3A4, GU*) or 0.5 µM (*CYP1A1, CYP1A2*) primers. The most suitable housekeeping gene for normalisation in LS180 cells was identified using geNorm (version 3.4, Center for Medical Genetics, Ghent, Belgium), which determines the most stable reference gene from a set of tested genes in a given cDNA sample panel [30]. Among a panel of 7 housekeeping genes tested, *glucuronidase β* (*GU*) proved to be the most stable gene in LS180 cells under the selected experimental conditions. Data were evaluated via calibrator-normalised relative quantification with efficiency correction using the LightCycler^®^ 480 software version 1.5.1.62 (Roche Applied Science, Mannheim, Germany). Results were expressed as the target/reference ratio divided by the target/reference ratio of the calibrator. The results are therefore corrected for sample inhomogeneities and variance caused by detection. All samples were amplified in duplicate and the mean of the technical duplicate was used for further calculation.

### 2.9. Statistical Analyses

Data were analysed using GraphPad Prism Version 8.43 and InStat Version 3.06 (GraphPad Software, San Diego, CA, USA). IC_50_ values were calculated using the four-parameter fit (sigmoidal dose-response curves with variable slope). Differences between mRNA expressions were tested using ANOVA with Dunnett’s post hoc test. Differences between the IC_50_ values in the growth inhibition assays were tested with the Kruskal–Wallis test with Dunn’s multiple comparisons posthoc test. A *p*-value < 0.05 was considered significant.

## 3. Results

### 3.1. Inhibition of Drug Transporters by Hydroxychloroquine

**Figure 1:**
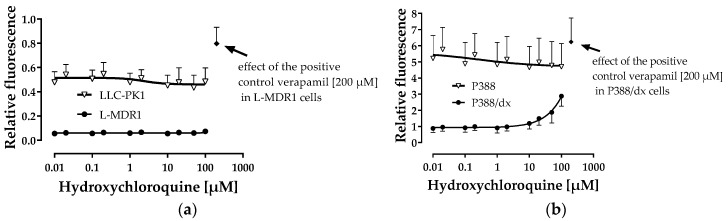
P-glycoprotein (P-gp) inhibition assay. Effect of hydroxychloroquine on intracellular calcein fluorescence in P-gp over-expressing L-MDR1 cells and the corresponding parental cell line LLC-PK1 (**a**) and in P-gp overexpressing P388/dx and the corresponding parental P388 cells (**b**). Results are depicted as mean ± S.D. of four independent experiments with each concentration tested in octuplet. For clarity, the results of the negative controls are not integrated into the figure: Buffer only yielded relative fluorescence values of 0.5 ± 0.009 in LLC-PK1 cells, 0.06 ± 0.007 in L-MDR1 cells, 6.2 ± 0.9 in P388 cells and 1.0 ± 0.2 in P388/dx cells. None of the hydroxychloroquine concentrations tested was cytotoxic as assessed within the cytotoxicity assay described in 2.2.

Hydroxychloroquine did not affect intracellular calcein fluorescence in L-MDR1 cells (over-expressing P-gp) and the corresponding parental cell line LLC-PK1 excluding potent P-gp inhibitory effects. We also tested the effects in P388/dx cells compared to P388 cells, because this system is more suitable to detect weak P-gp inhibitors. In P388/dx cells, but not in the parental cell line P388, intracellular calcein fluorescence was increased by hydroxychloroquine at concentrations higher than 10 µM clearly indicating weak P-gp inhibition. However, an IC_50_ value could not be calculated due to missing plateau effects (Figure 1b).

**Figure 2:**
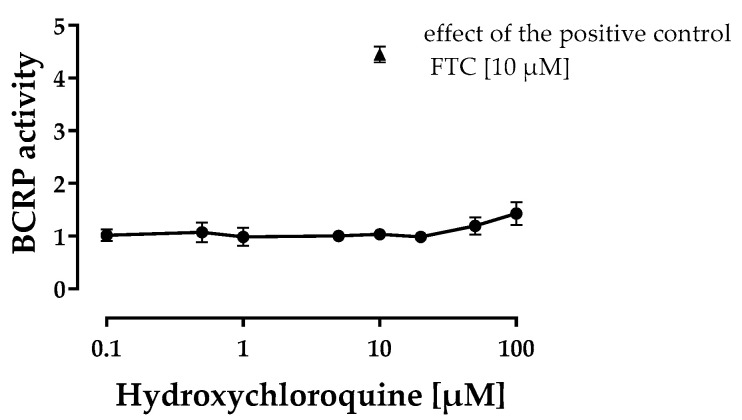
Breast cancer resistance protein (BCRP) inhibition assay. Effect of hydroxychloroquine on intracellular pheophorbide A fluorescence in MDKC-BCRP cells normalised to MDCKII cells. Results are depicted as mean ± S.D. with *n* = 3 biological replicates. None of the hydroxychloroquine concentrations tested was cytotoxic as assessed within the cytotoxicity assay described in 2.2.

Hydroxychloroquine slightly increased intracellular pheophorbide A fluorescence in MDCKII-BCRP cells but not in MDCKII cells at concentrations ≥ 50 µM indicating weak BCRP inhibition (Figure 2). In comparison to the positive control FTC, this inhibition was, however, negligible.

Hydroxychloroquine did not inhibit the uptake transporter OATP1B1, OATP1B3, and OATP2B1: In contrast to the positive control 20 µM rifampicin (inhibiting OATP1B1 by 80% and OATP1B3 by 90%), hydroxychloroquine did not decrease intracellular 8-FcA concentration in OATP1B1- or OATP1B3 over-expressing HEK293 cells, indicating the absence of OATP1B1 and OATP1B3 inhibition. Moreover, in contrast to the positive control (1 mM naringin, inhibiting OATP2B1 activity by 70%), hydroxychloroquine did not affect DBF uptake indicating the absence of OATP2B1 inhibition (data not shown).

### 3.2. P-gp and BCRP Substrate Characteristics of Hydroxychloroquine

**Figure 3:**
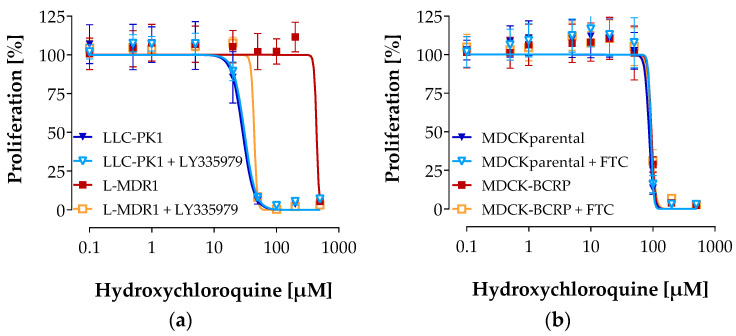
Growth inhibition assay. Concentration-dependent effect of hydroxychloroquine on the proliferation of the P-gp over-expressing cell line L-MDR1, the corresponding parental cell line LLC-PK1 with and without the specific P-gp inhibitor LY335979 (**a**) and of the BCRP over-expressing cell line MDCKII-BCRP and the corresponding parental cell line MDCKII with and without the specific inhibitor fumitremorgin C (FTC) (**b**). Each curve depicts the results of four experiments with each concentration tested in octuplet. Data are expressed as mean ± S.D. for *n* = 32 wells.

The potency of hydroxychloroquine to inhibit cell growth significantly differed in P-gp over-expressing cells (L-MDR1) compared to the parental cell line (LLC-PK1) (IC_50_: 82 ± 10 µM for LLC-PK1; 427 ± 16 µM for L-MDR1; *p* < 0.001). L-MDR1 cells were about 5-fold more resistant to the anti-proliferative effects of hydroxychloroquine (*p* < 0.01, Figure 3a). Moreover, this resistance was abolished in the presence of the strong and specific P-gp inhibitor LY335979 (zosuquidar), whereas no effect was observed in the parental cell line lacking P-gp (IC_50_: 93 ± 2 µM for LLC-PK1 + LY335797; 90 ± 1 µM for L-MDR1 + LY335979; *p* > 0.05). Together, this verifies that the increased resistance of L-MDR1 cells to hydroxychloroquine can be attributed to P-gp over-expression. In summary, these results clearly indicate that hydroxychloroquine is a substrate of the efflux transporter P-gp.

In contrast, there was no difference between the potency of hydroxychloroquine to inhibit cell growth in the BCRP over-expressing cell line MDCKII-BCRP and the parental cell line MDCKII with or without the potent and specific BCRP inhibitor FTC (Figure 3b; IC_50_: 89 ± 12 µM for MDCKII; 89 ± 7 µM for MDCKII + FTC; 88 ± 9 µM for MDCKII-BCRP, and 92 ± 5 µM for MDCKII-BCRP + FTC, *p* > 0.05). These experiments indicate that hydroxychloroquine is not transported by BCRP.

### 3.3. Induction of PXR and AhR Regulated Genes by Hydroxychloroquine

**Figure 4:**
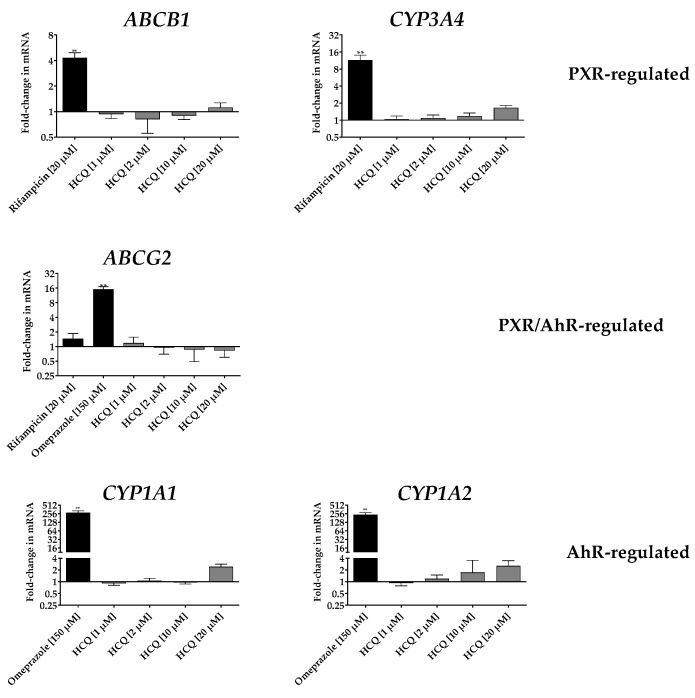
Effects of hydroxychloroquine (HCQ) on mRNA expression in LS180 cells compared to untreated medium control after 4 days of incubation. Rifampicin (20 µM) served as a positive control for pregnane X (PXR)-driven genes (*CYP3A4, ABCB1* and *ABCG2)* and omeprazole (150 µM) served as a positive control for AhR-driven genes (*ABCG2, CYP1A1, CYP1A2*). Expression data were normalised to the medium control and the housekeeping gene *glucuronidase β*. Data are expressed as mRNA changes ± S.D. for *n* = 5 biological replicates. Data were analysed using ANOVA with Dunnett’s post hoc test compared to the medium control. ** *p* < 0.01.

Possible inductions of PXR-regulated (*ABCB1, CYP3A4, ABCG2*) and AhR-regulated (*ABCG2, CYP1A1, CYP1A2*) genes by hydroxychloroquine were tested in LS180 cells. As expected, the positive control rifampicin (PXR ligand) significantly up-regulated the prototypical PXR-driven genes *CYP3A4* and *ABCB1* and also slightly the *ABCG2* gene regulated by both, PXR and AhR, which was however not relevant compared to the profound effects of the positive control omeprazole. Omeprazole (AhR ligand) significantly up-regulated mRNA expression of the AhR-regulated genes *ABCG2, CYP1A1*, and *CYP1A2*. In contrast, hydroxychloroquine had no significant effect on the expression of any of these genes (Figure 4).

## 4. Discussion

Hydroxychloroquine is licensed for the treatment and prophylaxis of malaria and the treatment of various autoimmune diseases. In addition, it is explored and considered for the treatment of several bacterial, fungal, and viral infections including COVID-19 [3,5,6,7,35]. The pharmacodynamic interaction potential of hydroxychloroquine is mainly restricted to its well-known QT-prolonging and pro-arrhythmic effects [3,4,7]. In contrast, its pharmacokinetic drug-drug interaction potential has never been studied in detail, although hydroxychloroquine has been on the market for decades. So far, isolated case reports suggest that hydroxychloroquine increases digoxin plasma concentrations through inhibition of P-gp [16], but whether hydroxychloroquine actually inhibits this important efflux transporter has never been investigated. We, therefore, had a closer look at the possible transporter-based drug-drug interaction potential of hydroxychloroquine and focused on transporters frequently implicated in such pharmacokinetic interactions.

Hydroxychloroquine represents a drug with a very high volume of distribution (5522 L in blood; 44,257 L in plasma) and due to its accumulation in blood cells, quantification of whole blood concentrations rather than plasma concentrations is recommended [36,37]. At steady-state (400 mg/day), blood concentrations up to 9 µM have been measured [38] and in SLE patients the proposed target blood concentration is 1000 ng/mL (=3 µM) [39]. Thus, evaluating hydroxychloroquine concentration in the µM range seems appropriate and the effects of hydroxychloroquine concentrations in the µM range must be considered clinically meaningful. Due to the accumulation in tissue/cells, the average intracellular concentration at steady-state is postulated to be 1 mM and even 80 mM within lysosomes [37,40]. Interestingly, the mainly plasma membrane-located drug transporter P-gp is also present in the lysosomal membrane actively sequestering P-gp substrates into lysosomes [41]. Our data for the first time indicate that hydroxychloroquine is a P-gp substrate: cells with P-gp over-expression were much more resistant towards this compound than the corresponding parental cells and this resistance was abolished in the presence of the specific P-gp inhibitor LY335979 (Figure 3a). Thus, hydroxychloroquine accumulation in lysosomes might not only be driven by low lysosomal pH, but also by active transport via P-gp. Moreover, based on the assumption that hydroxychloroquine is a P-gp substrate, diminished intracellular concentration of hydroxychloroquine might also have contributed to the poor response in SLE patients with high P-gp activity in the cell membrane of leucocytes. These patients received a combination of prednisolone and hydroxychloroquine and originally the poor response in patients with high P-gp activity was attributed to corticosteroid resistance [42].

Our data also indicate that hydroxychloroquine inhibits P-gp at extracellular concentrations exceeding 10 µM. Together with the assumption that it is a P-gp substrate, this inhibition is most likely competitive. Recently, another mechanism for P-gp inhibition has been demonstrated: in cancer cells, hydroxychloroquine inactivates P-gp located in lysosomes by increasing lysosomal pH (after 12 h of incubation), thus sensitising cells to chemotherapeutic P-gp substrates such as doxorubicin [43]. However, the calcein assay applied in our study measures short-term effects (total incubation time only 45 min in the P388 cell system); thus, it can be excluded that the effect observed can be attributed to a (sub-acute) increase in lysosomal pH. In fact, direct P-gp inhibition may also have contributed to the observed increased sensitivity of cells towards doxorubicin described previously [43]. In addition, the clinical interaction between the prototypical P-gp substrate digoxin and hydroxychloroquine also argues for P-gp inhibitory potential of hydroxychloroquine: Two case reports demonstrated increased plasma concentrations of digoxin during concurrent therapy with hydroxychloroquine [16], indicating intestinal P-gp inhibition. According to the formula published by Zhang and co-workers [44], after a dose of 200/400 mg hydroxychloroquine, intestinal concentrations are expected to reach nearly 2/4 mM, which is obviously high enough for potent P-gp inhibition and thus increased absorption and potential toxicity of digoxin. Whether P-gp inhibition by hydroxychloroquine is clinically relevant for other P-gp substrates with a narrow therapeutic index (e.g., dabigatran etexilate) remains to be investigated.

Beyond efflux transporters, several uptake transporters can also mediate drug-drug interactions, especially members of the OATP family [11,45]. Previous data demonstrated weak inhibition of organic anion transporters (OATs) by hydroxychloroquine, but no inhibition of organic cation transporters (OCTs) and organic cation/carnitine transporters (OCTNs) [46]. Our data expand these findings and clearly rule out that hydroxychloroquine (up to 100 µM) inhibits OATP1B1, OATP1B3, and OATP2B1, underlining previous data on OATP1B1 [46]. In contrast, the latter study found weak inhibition of OATP1B3 and OATP2B1 (about 20% at a concentration of 10 µM). However, this discrepancy to our data might be explained by the usage of other cell lines, other substrates, and the lack of normalisation to a parental cell line as performed in the previous study. Moreover, there are no reports on clinically relevant interactions with OATP1B3 or OATP2B1 substrates, rendering relevant inhibition of these transporters by hydroxychloroquine unlikely. Interestingly, hydroxychloroquine seems to be a competitive inhibitor of OATP1A2 and inhibits the uptake of the OATP1A2 substrate all-*trans*-retinol in vitro [46]. The authors of this finding postulated that this inhibition might contribute to the retinal degeneration observed in patients under hydroxychloroquine therapy.

Apart from inhibition, induction of drug transporters or drug-metabolising enzymes can cause substantial drug-drug interactions, which are frequently mediated by the nuclear receptor PXR and sometimes by AhR [13,47,48]. We, therefore, investigated whether hydroxychloroquine induces typical genes regulated by these transcription factors. Because the mRNA expression of none of these genes was significantly influenced, hydroxychloroquine is not a PXR or AhR ligand and unlikely acts as a perpetrator in drug-drug interactions based on transcriptional induction mediated by PXR or AhR.

Limitations: (1) We did not test the metabolites of hydroxychloroquine, which might also contribute to its drug-drug interaction potential. (2) We limited ourselves to the investigation of drug transporters most relevant for drug-drug interactions. (3) In the induction experiment, we restricted the analysis to marker genes of induction mediated by PXR or AhR. (4) We used the growth inhibition assay for assessing substrate characteristics. Although this is an indirect assay not measuring the transport itself, obtained data are congruent, unambiguous and clearly indicate that hydroxychloroquin is a P-gp substrate.

## 5. Conclusions

Our data suggest that hydroxychloroquine is a substrate and weak inhibitor of P-gp. In contrast, it is not a substrate of BCRP and does not inhibit this efflux transporter or the uptake transporters OATP1B1, 1B3, and 2B1. Moreover, genes regulated by PXR or AhR are not induced by hydroxychloroquine. Taken together, this in vitro study for the first time demonstrates that the transporter-based interaction potential of hydroxychloroquine appears to be low. Only P-gp mediated interactions should be studied in more detail in clinical studies, particularly if the oral bioavailability of a compound is significantly restricted by this transporter (e.g., dabigatran etexilate). Until clinical relevance is known, administration of hydroxychloroquine and P-gp substrates should be separated in time by hours and, as a precaution, hydroxychloroquine should not be administered until absorption of the P-gp substrate is largely complete.

## Figures and Tables

**Table 1:** Primer sequences and annealing temperatures.

Gene	Forward Primer 5′-3′	Reverse Primer 5′-3′	A [°C]	Ref.
*ABCB1*	CCCATCATTGCAATAGCAGG	TGTTCAAACTTCTGCTCCTG	60	[29]
*ABCG2*	AGATGGGTTTCCAAGCGTTCAT	CCAGTCCCAGTACGACTGTGACA	57	[29]
*CYP1A1*	TCCGGGACATCACAGACAGC	ACCCTGGGGTTCATCACCAA	65	[31]
*CYP1A2*	CATCCCCACAGCACAACAA	TCCCACTTGGCCAGGACTTC	63	[32]
*CYP3A4*	TTCAGCAAGAAGAACAA	GGTTGAAGAAGTCTCTAAGC	57	[33]
*GU*	TTCACCAGGATCCACCTCTG	AGCACTCTCGTCGGTGACTG	61	[34]

A = annealing temperature.
